# The Use of Machine Learning Approaches for the Diagnosis of Acute Appendicitis

**DOI:** 10.1155/2020/7306435

**Published:** 2020-04-25

**Authors:** Omer F. Akmese, Gul Dogan, Hakan Kor, Hasan Erbay, Emre Demir

**Affiliations:** ^1^Department of Computer Technologies, University of Hitit, University of Kırıkkale, Çorum 19500, Turkey; ^2^Department of Surgical Medical Sciences, University of Hitit, Çorum 19040, Turkey; ^3^Department of Computer Technologies, University of Hitit, Çorum 19300, Turkey; ^4^Department of Computer Engineering, University of Turkish Aeronautical Association, Ankara 06790, Turkey; ^5^Department of Biostatistics, University of Hitit, Çorum 19040, Turkey

## Abstract

Acute appendicitis is one of the most common emergency diseases in general surgery clinics. It is more common, especially between the ages of 10 and 30 years. Additionally, approximately 7% of the entire population is diagnosed with acute appendicitis at some time in their lives and requires surgery. The study aims to develop an easy, fast, and accurate estimation method for early acute appendicitis diagnosis using machine learning algorithms. Retrospective clinical records were analyzed with predictive data mining models. The predictive success of the models obtained by various machine learning algorithms was compared. A total of 595 clinical records were used in the study, including 348 males (58.49%) and 247 females (41.51%). It was found that the gradient boosted trees algorithm achieves the best success with an accurate prediction success of 95.31%. In this study, an estimation method based on machine learning was developed to identify individuals with acute appendicitis. It is thought that this method will benefit patients with signs of appendicitis, especially in emergency departments in hospitals.

## 1. Introduction

Acute appendicitis occurs in a wide range of patients of all ages. It is common especially in young adults between the ages of 10 and 30. In addition, about 7% of the population suffers from appendicitis throughout their lives [1–3]. Appendectomy for acute appendicitis is one of the most common emergency surgical procedures performed by general surgeons. Since delayed surgery can increase the rate of perforated appendicitis, appendectomy must be performed a few hours after diagnosis [4–7]. Although appendicitis is the most common surgical pathology of the abdomen, its diagnosis, follow-up, and treatment vary according to the surgeon's experience and preference [8, 9]. Approximately one-third of patients present with atypical complaints [10]. This condition is often associated with complications and delays in diagnosis. Many patients suspected of acute appendicitis develop a complication of 22% to 62%, even if they are diagnosed and operated on without any complications [11]. A definitive diagnosis is essential to prevent complications of appendicitis and to prevent negative appendectomy. The diagnosis of appendicitis is based primarily on biochemical tests that are decisive for inflammation. The use of this method alone revealed a negative appendectomy rate of 12.3–19%. Despite the remarkable improvements in modern radiography imaging and diagnostic laboratory examinations, the correct diagnosis of acute appendicitis remains a challenge [12–15]. In order to make the diagnosis without any complications, studies were performed with easily accessible methods such as CRP (C-reactive protein), leukocyte count, neutrophil ratio, bilirubin, multislice computed tomography, and ultrasonography imaging techniques [16, 17]. Radiological methods, especially ultrasonography and computed tomography, are widely and successfully used in the diagnosis of acute appendicitis and its complications [18, 19]. However, since these methods require specialized equipment and experienced radiologists, data mining, a different method for diagnosing the disease, has been used. Data mining is a methodology to discover hidden patterns from large datasets using statistical approaches [20]. Unlike traditional statistical techniques, data mining is mainly concerned with transforming data into information and learning from that data [21]. Among the various applications of data mining, its use in the health field is no exception. It has proven to be very useful for analyzing medical datasets and extracting strong molds [22, 23]. In this article, we tried to estimate the necessity of surgery by using data from blood samples of patients. Thus, it is aimed to test the accuracy of the diagnosis related to the disease to minimize the resource consumption and to provide more accurate utilization of the limited health services.

In most patients suspected of acute appendicitis, diagnostic procedures are performed using blood values or images obtained. In the study, which investigated the relationship between basic statistical methods and acute appendicitis, the patients who underwent 302 appendectomies in 2008 were examined for gender, age, and leukocyte values [24].

There are few studies on predicting or treating acute appendicitis with data mining using computers. Demirhan et al. conducted a survey of artificial intelligence applications in medicine. A system has been developed to evaluate the role of artificial neural networks (ANNs) in the diagnosis of patients with acute right groin pain. In the developed system, patient data collected from a hospital were used in the training and testing of ANN. The performance of the system was compared with the Alvarado score and the evaluations of experienced doctors. The patient's symptoms and signs, hematological assessment, and demographic information were used as input data in the ANN [25].

In another study, the performance of fuzzy inference systems and logistic regression classifiers was compared using appendicitis data [26]. In the study conducted on samples selected from the KEEL (Knowledge Extraction based on Evolutionary Learning) database with the R program, the accuracy values of the results obtained from fuzzy inference systems and logistic regression were high. In another study where data were processed by the clustering method, gender, abdominal pain, age parameters and leukocyte, platelet, lymphocyte, neutrophil, and C-reactive protein (CRP) values were used. The number of ideal clusters in the study was determined as four. This clustering study was intended to design an ideal decision support system, including physician views [27].

## 2. Materials and Methods

Machine learning is the term automatic data analysis for analytical and statistical pattern recognition and modeling. It allows recursively learning from the new data, updating the models that have been created, and thus finding hidden information or patterns in large datasets. Data mining focuses on the exploration of previously unknown features of an extensive existing dataset [28]. Data mining methods are used by many researchers for prediction purposes. The classification and evaluation under data mining techniques help in the creation of training data, the classification of the estimation model, and the testing of classification efficiency [29]. It can be ensured that the models can be adapted to future datasets.

In this study, the CRISP-DM methodology was applied for data mining. The CRISP-DM methodology of data mining proposes step-by-step procedures so that the process can be reliable and standard. This methodology follows a cyclical process that includes business understanding, data understanding, data preparation, modeling, evaluation, and deployment [30].

In data mining, models can be classified under two main headings: predictive and descriptive. In predictive models, a model is developed by acting on the results of known data. It is intended to estimate the results for unknown data, that is, make inferences that will predict the future. However, in descriptive models, it is provided to define the patterns in the available data that can be used to guide decision making [31].

This study was carried out with the data of clinical records collected at the Hitit University Training and Research Hospital. These data show records of gender, laboratory markers, and surgical conditions belonging to patients applied to the hospital with suspicion of appendicitis. In the design of the study, blood samples of 595 patients admitted to Hitit University Training and Research Hospital were taken. Those diagnosed with acute appendicitis and who underwent surgery were identified as one group; others were identified as a separate group. In addition to descriptive statistical analysis, machine learning methods have been used to predict groups. Before starting the research, priori power analysis was performed using *t*-test in independent groups. In order to reach 95% power with *α* = 0.05 error, the power analysis found using Cohen *d* = 0.35 effect size calculated using the literature information, and it was decided to include a total of 428 patients, 214 in each group.

All procedures performed in studies involving human participants were in accordance with the ethical standards of the institutional and national research committee (the Ethics Committee of Noninterventional Research in Hitit University (reference number: 2019-45)) and with the 1964 Helsinki declaration and its later amendments or comparable ethical standards. Informed consent was obtained from all individuals included in this study.

### 2.1. Data and Statistical Analysis

Independent sample *t*-test was used for parametric test assumptions in numerical measurement comparisons in groups with and without surgery. Pearson correlation analysis and chi-square tests were used for weighting. Machine learning algorithms were used for disease classification. Classification success was evaluated with accuracy, precision, and recall results. Both qualitative and quantitative data were analyzed by machine learning algorithms. In the study, data analyses were carried out using SPSS (22.0), Rapidminer (9.5) packet programs, and Python (3.7) programming language.

When Table 1 is examined, the data were summarized according to the gender of the patients, their operating status, and blood test results. While gender and blood test results are used as input data, the group variable is determined as the target (dependent) variable.

Table 2 shows the blood test results from the dataset. In the variable Group, 0 refers to those without surgery and 1 refers to those who were operated on. For the gender variable, 1 refers to females and 2 refers to males. Other data are the values in the blood samples. Values are presented as mean ± standard deviation. Bold denotes a significant *p* value less than 0.05. *p* values are given in Table 2. Only values less than one-thousand were shown as *p* < 0.001 in accordance with APA reporting.

In this study, the attributes and doctor's recommendations, which are stated as important for diagnosis in the literature, were taken into consideration, and the weights of the qualities were calculated. The relationship between two or more variables has not been investigated. The weights of numerical and categorical variables in predicting a categorical outcome variable were determined. The chi-square weighting method was used to determine these weights. The higher the weight of an attribute, the more relevant it is considered. Calculated weights were normalized from 0 to 1.  Table 3 shows the attributes and weightings.

When the analysis of the established model is performed, the performance of the measurements obtained is evaluated. In the calculation of these values, true positive, false positive, true negative, and false negative values are used.

TP (correct decision): in the actual case, it means the accurate estimate of the patients who were operated on.

FP (type I error): patients who were not operated on in the real situation were defined as operated.

TN (correct decision): it means the accurate estimation of patients who do not undergo surgery in real condition.

FN (type II error): patients who underwent surgery in real condition were defined as not operated.

**Accuracy:** the ratio of correctly estimated samples to the number of all samples. That is, the test is the rate of total correct diagnoses.

(1) $$\begin{array}{c} \text{Accuracy}=\frac{\text{TP}+\text{TN}}{\text{TP}+\text{FP}+\text{TN}+\text{FN}}=\frac{\text{TP}+\text{TN}}{\text{P}+\text{N}}. \end{array}$$

**Precision (positive predictive value):** it is the ratio of correctly predicted positive samples to the number of samples estimated in the positive class.

(2) $$\begin{array}{c} \text{Precision}=\frac{\text{TP}}{\text{TP}+\text{FP}}=\frac{\text{TP}}{{\text{P}}^{\prime }}. \end{array}$$

**Recall (sensitivity, true positive rate):** it is the ratio of correctly predicted positive samples to the number of samples in the true positive class.

(3) $$\begin{array}{c} \text{Recall}=\frac{\text{TP}}{\text{TP}+\text{FN}}=\frac{\text{TP}}{\text{P}}. \end{array}$$

**Figure 1:**
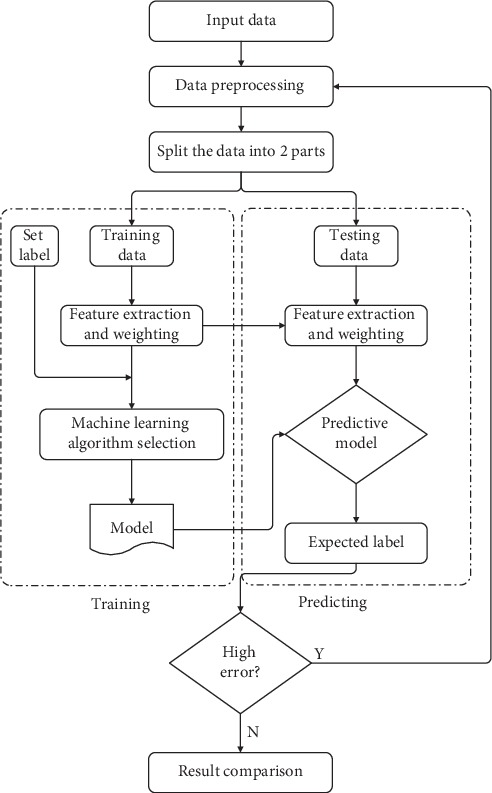
The architecture of the proposed system.

Predictive data mining was performed by machine learning algorithms, and the accuracy of these methods was compared. In the models, some of the data were used as training data, and some were used to test the accuracy of the model. The proposed architecture is shown in Figure 1.

The data collected according to the architecture seen in Figure 1 were subjected to preprocessing. The quality of the data dramatically influences the outcome of the estimation. This means that preprocessing plays an important role in the model [32]. After determining the dependent variable, the data were divided into two groups as training and test data. Seventy percent of the data was used as training data, and thirty percent was used for testing the model. Finally, the performance of the model was evaluated. The missing values in the records have been deleted to maintain the accuracy of the attributes. In the preprocessing stage, the missing data have been cleared; as a result, the number of clinical records decreased from 595 records to 428.

**Figure 2:**
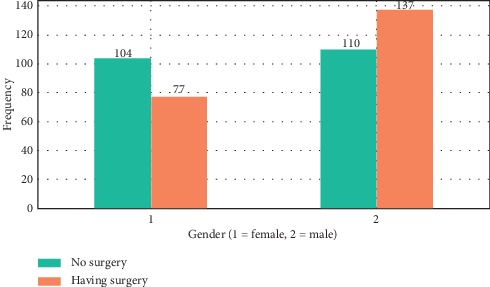
Graph of those who underwent surgery according to gender after data preprocessing.

According to Figure 2, there were 77 (36%) females and 137 (64%) males in the having surgery group and 104 (48.6%) females and 110 (51.4%) males in the no surgery group. In addition, half of the 428 records were female, and half were male.

It was found that the “gradient boosted trees” algorithm achieves the best success with an accurate prediction success rate of 95.31%. As a general principle, boosting algorithms try to achieve a strong learner by combining the weak learner obtained in each iteration under specific rules. The basic idea is to minimize the error and determine the target outputs for the next model. This technique is based on the advancement of subsequent estimates by learning from previous prediction errors.

It is a machine learning technique for gradient boosting, regression, and classification problems. Each tree is grown using information from previously created trees. The basic algorithm can be generalized to the following where *x* represents features and *y* represents response:

1. Gradient boosting creates a *f*_1_ function that generates predictions in the first iteration.

(4) $$\begin{array}{c} f_{1}\left(x\right)=y. \end{array}$$

(2) It calculates the difference between the estimates and the target value and creates the function *h*_1_ for these differences.

(5) $$\begin{array}{c} h_{1}\left(x\right)=y-\;f_{1}\left(x\right). \end{array}$$

(3) It creates a new tree.

(6) $$\begin{array}{c} f_{2}\left(x\right)=\;f_{1}\left(x\right)+\;h_{1}\left(x\right). \end{array}$$

(4) It recalculates the difference between estimates and goals.

(7) $$\begin{array}{c} h_{2}\left(x\right)=y-\;f_{2}\left(x\right). \end{array}$$

(5) In this way, it continually tries to increase the success of the “*F*” function and to reduce the difference between predictions and targets to zero.

Gradient descent is a very general optimization algorithm that can find optimal solutions to a wide range of problems. Estimates are updated so that the loss function (MSE) is minimum, and the estimated values are close to the actual values. The general idea of the gradient descent is to adjust the parameters repeatedly to minimize a cost function.

(8) $$\begin{array}{c} J\left(\theta \right)=\frac{1}{n}\overset{n}{\underset{i=1}{\sum }}{\left(h_{\theta }\left(x^{\left(i\right)}\right)-y^{\left(i\right)}\right)}^{2}, \end{array}$$

where *n* is the number of training examples, *x*^(*i*)^ is the input vector for the *i*^th^ training examples, *y*^(*i*)^ is the class label for the *i*^th^ training examples, *θ* is the chosen parameter values or weights, and *h*_*θ*_(*x*^(*i*)^) is the algorithm's prediction for the *i*^th^ training examples using the parameters *θ*.

At each iteration, the residue of the loss function is calculated using the gradient descent method and becomes the target value for the next iteration [33]. In summary, gradient boosting uses the gradient descent method to minimize the derivable loss function value of each weak classifier.

## 3. Results and Discussion

**Figure 3:**
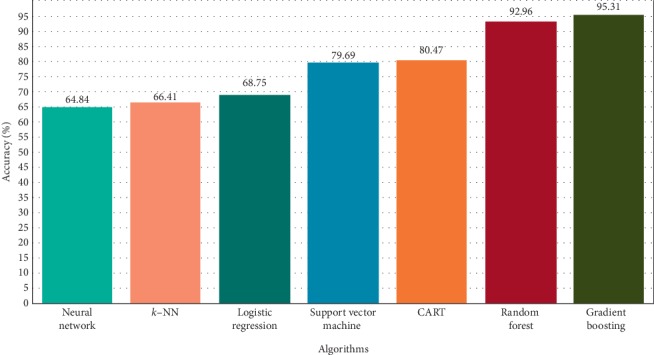
Accuracy percentages of algorithms.

During the research, 7 machine learning algorithms were tried. The most successful algorithm was gradient boosted trees. The accuracy rates of the algorithms applied in the research process are shown in Figure 3.

After the data preprocessing step, 70% of the clinical records falling to 428 were used as training data. The model was tested with 30% of the data. Of the 128 clinical records, 122 were correctly estimated. Thus, the predictive accuracy of the model was 95.31%.

**Figure 4:**
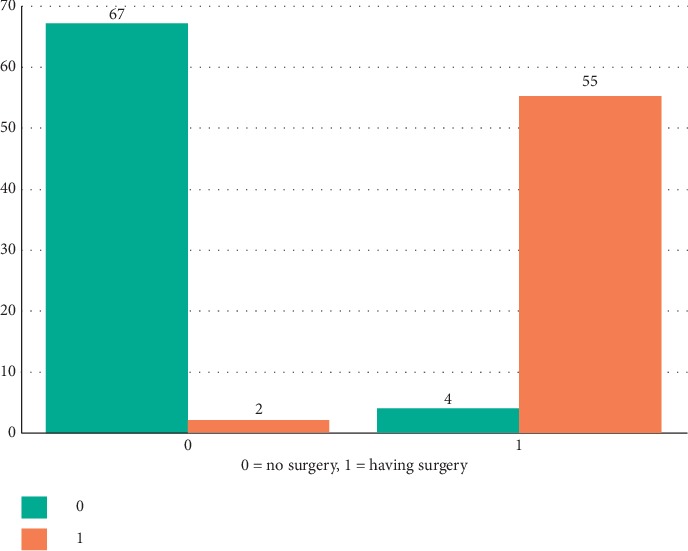
Gradient boosting tree prediction.

Figure 4 shows the estimation results according to the gradient boosted trees algorithm. Accordingly, 67 out of 69 patients without surgery were correctly estimated. Of the 59 patients who underwent surgery, 55 were correctly estimated.

The ratio of correctly estimated samples to the number of all samples according to Table 4 is 95.31%. This ratio represents the total accuracy. It is represented as TP (true positives), TN (true negatives), FN (false negatives), and FP (false positives).

Examination and simple laboratory tests can often diagnose acute appendicitis, but when signs and symptoms are atypical, diagnosis is difficult. It has been observed that researchers who carry out their studies using whole blood counts have more emphasis on neutrophil/lymphocyte rates in the diagnosis of acute appendicitis. In diagnostic studies, mostly computerized imaging techniques and statistical analysis of the values obtained from blood counts come to the forefront. With the use of various algorithms, the contribution of computer estimation skills is seen in a small number of studies.

In this retrospective study, we proposed a model that predicts the necessity of appendicitis surgery using laboratory data of patients aged 0–18 years. The proposed model has been developed as a diagnostic tool to improve surgical decision making in patients with suspected acute appendicitis. In this way, unnecessary appendectomies and possible postoperative complications can be prevented. Increased use of preoperative computed tomography (CT) for the diagnosis of acute appendicitis has helped to reduce negative appendectomy rates [34–37]. However, the use of CT has raised concerns about unnecessary exposure to radiation [38]. In addition to radiation exposure, these methods require specialized equipment and experienced radiologists. A different aspect of this study compared to others is the use of machine learning methods based on laboratory data for the diagnosis of appendectomy. The most important features that add value to the research globally can be listed as follows. The proposed model may assist in the diagnosis in the absence of specialized equipment and personnel in making decisions for surgical operation. In addition, the accuracy of disease-related diagnoses can be tested, resource consumption can be reduced, and limited health care can be used more accurately. In addition, an easy, fast, and accurate estimation method has been developed for the diagnosis of appendectomy which is the main problem of the article. Thus, accurate diagnosis can reduce hospital stay and cost. In future studies, new patient data should be added to the continuing education of the model. It is thought that the accuracy of estimation will increase as the number of data increases.

## 4. Conclusions

In this article, different machine learning methods were investigated in order to predict the necessity of appendicitis surgery by examining only the blood sample data of patients presenting with abdominal pain complaints. An easy, fast, and accurate estimation method has been developed for the diagnosis of appendectomy, which is the main problem of the article. Machine learning algorithms were used for patient data with suspicion of appendicitis, and the accuracy of these algorithms was compared. It was found that the gradient boosting tree algorithm achieves the best success with an accurate prediction success of 95.31%. The closest value to this result was found to be 92.96% with a random forest algorithm. In the study, the accuracy rate was considered as the most important factor.

## Figures and Tables

**Table 1:** Dataset description.

Name	Type	Description	Role
Group	Categorical	No surgery/having surgery	Target
Gender	Categorical	Female/male	Input
HGB	Numeric	Hemoglobin	Input
NEU	Numeric	Neutrophil	Input
LYM	Numeric	Lymphocytes	Input
MCV	Numeric	Mean corpuscular volume	Input
MPV	Numeric	Mean platelet volume	Input
HTC	Numeric	Hematocrit	Input
PLT	Numeric	Thrombosis	Input
CRP	Numeric	C-reactive protein	Input
WBC	Numeric	White blood cells (leukocytes)	Input

Categorical data represent types of data which may be divided into groups. Numerical data are data expressed in numbers, unlike letters or words. Target: dependent variable; input: independent variable.

**Table 2:** Blood test results in acute appendicitis dataset.

Name	Value range	Having surgery	No surgery	*p* value
Group	0 or 1			
Gender	1 or 2	77/137	104/110	0.008
HGB	1.4–130	13.63 ± 8.18	13.02 ± 1.43	0.348
NEU	0.8–29	11.82 ± 5.31	9.11 ± 5.77	**<0.001**
LYM	0.2–94	3.09 ± 7.09	2.44 ± 1.17	0.016
MCV	4–97.3	80.98 ± 8.76	81.20 ± 5.79	0.434
MPV	6–99	14.03 ± 20.02	9.31 ± 7.93	0.708
HTC	3–99	40.32 ± 6.24	39.69 ± 3.49	0.143
PLT	111–593	272.49 ± 73.58	274.37 ± 82.73	0.954
CRP	0–302	39.61 ± 51.62	22.86 ± 40.64	**<0.001**
WBC	6–31590	15280 ± 5302	12541 ± 6259	**<0.001**

Group: 0 refers to those without surgery and 1 refers to those who were operated on. Gender variable: 1 refers to females and 2 refers to males. HGB: hemoglobin, NEU: neutrophil, LYM: lymphocytes, MCV: mean corpuscular volume, MPV: mean platelet volume, HTC: hematocrit, PLT: thrombosis, CRP: C-reactive protein, and WBC: white blood cells (leukocytes). *p* values < 0.05 are statistically significant. Bold denotes a significant *p* value less than 0.05.

**Table 3:** Attributes and weighting.

No		Attributes	Weighting (chi-square)
1		NEU	1
2		WBC	0.994
3		CRP	0.436
4		MPV	0.201
5		LYM	0.120
6		HTC	0.119
7		Gender	0.079
8		MCV	0.075
9		MPV	0.049
10		HB	0

HGB: hemoglobin, NEU: neutrophil, LYM: lymphocytes, MCV: mean corpuscular volume, MPV: mean platelet volume, HTC: hematocrit, PLT: thrombosis, CRP: C-reactive protein, and WBC: white blood cells (leukocytes).

**Table 4:** Results of gradient boosting tree analysis.

Accuracy: 95.31%	True 1	True 0	Total	Class precision (%)
Pred. 1	55 (TP) Correct Decision	2 (FP) Type I error	57 (P′)	96.49
Pred. 0	4 (FN) Type II error	67 (TN) Correct Decision	71 (N′)	94.36
Total	59 (P)	69 (N)	128 (P + N)	
Class recall	93.22%	97.10%		

TP: true positives, TN: true negatives, FN: false negatives, and FP: false positives. Precision: it is the ratio of correctly predicted positive samples to the number of samples estimated in the positive class. Recall: it is the ratio of correctly predicted positive samples the ratio to the number of samples in the true positive class.

## Data Availability

Full collected data can be obtained through email from the corresponding author.
